# Supplementary material for: Processing oxidatively damaged bases at DNA strand breaks by APE1
Source: Nucleic Acids Res. 2022 Aug 26;50(16):9521–33. doi: 10.1093/nar/gkac695 (PMC9458457; doi:10.1093/nar/gkac695)
Supplement: gkac695_Supplemental_File [file gkac695_supplemental_file.pdf]

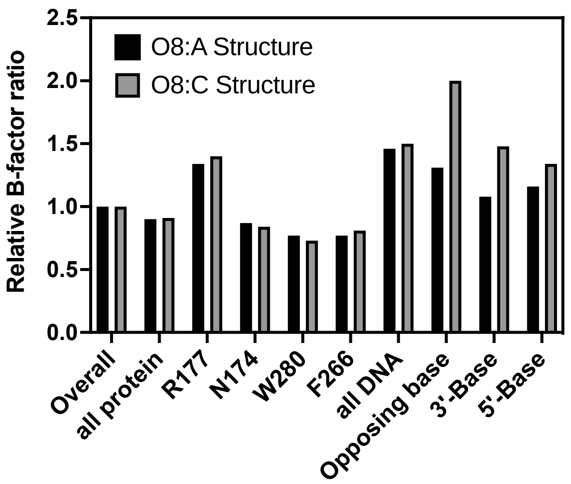

Supplemental Figure 1: Relative B-factors for the 3’-8-oxoG exonuclease structures broken down by structural region/residue. B-factors for each indicated region or residue are normalized to the overall B-factor for the corresponding structure.

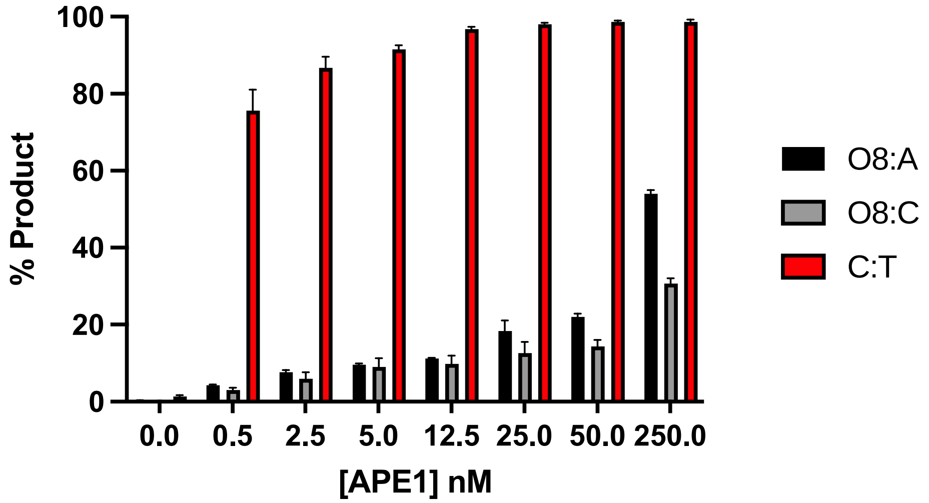

Supplemental Figure 2: Formation of the APE1 5' to 3' exo cleavage product over a period of 30 min at 37°C for a range of WT APE1 concentrations from 0 to 250 nM APE1 with O8:A (black bars), O8:C (grey bars), and C:T (red) base parings at the 3'-end of the nick. Raw data is in Supplemental Figure 3.

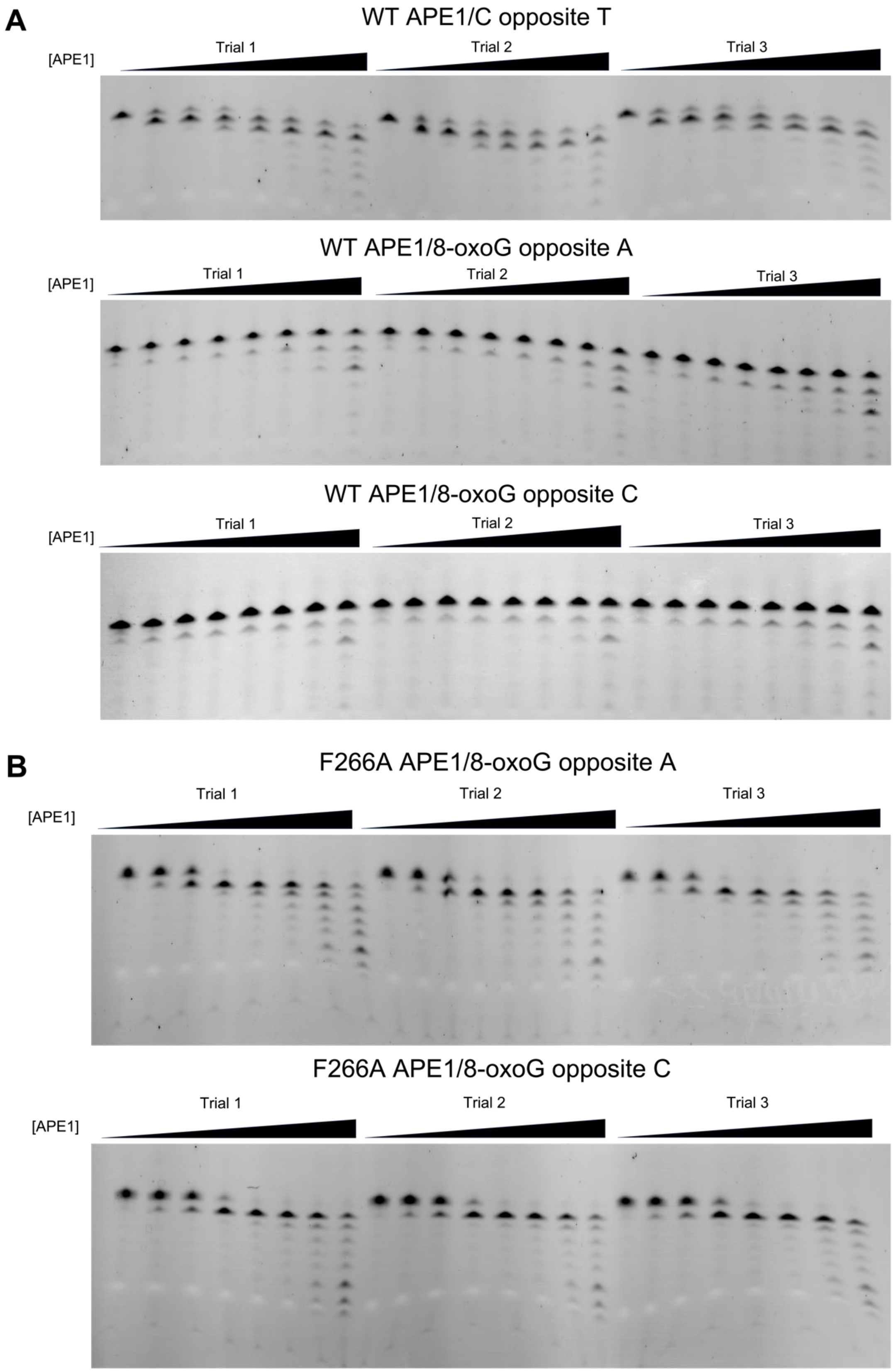
A
WT APE1/C opposite T
[APE1]
Trial 1
Trial 2
Trial 3
WT APE1/8-oxoG opposite A
[APE1]
Trial 1
Trial 2
Trial 3
WT APE1/8-oxoG opposite C
[APE1]
Trial 1
Trial 2
Trial 3
B
F266A APE1/8-oxoG opposite A
[APE1]
Trial 1
Trial 2
Trial 3
F266A APE1/8-oxoG opposite C
[APE1]
Trial 1
Trial 2
Trial 3

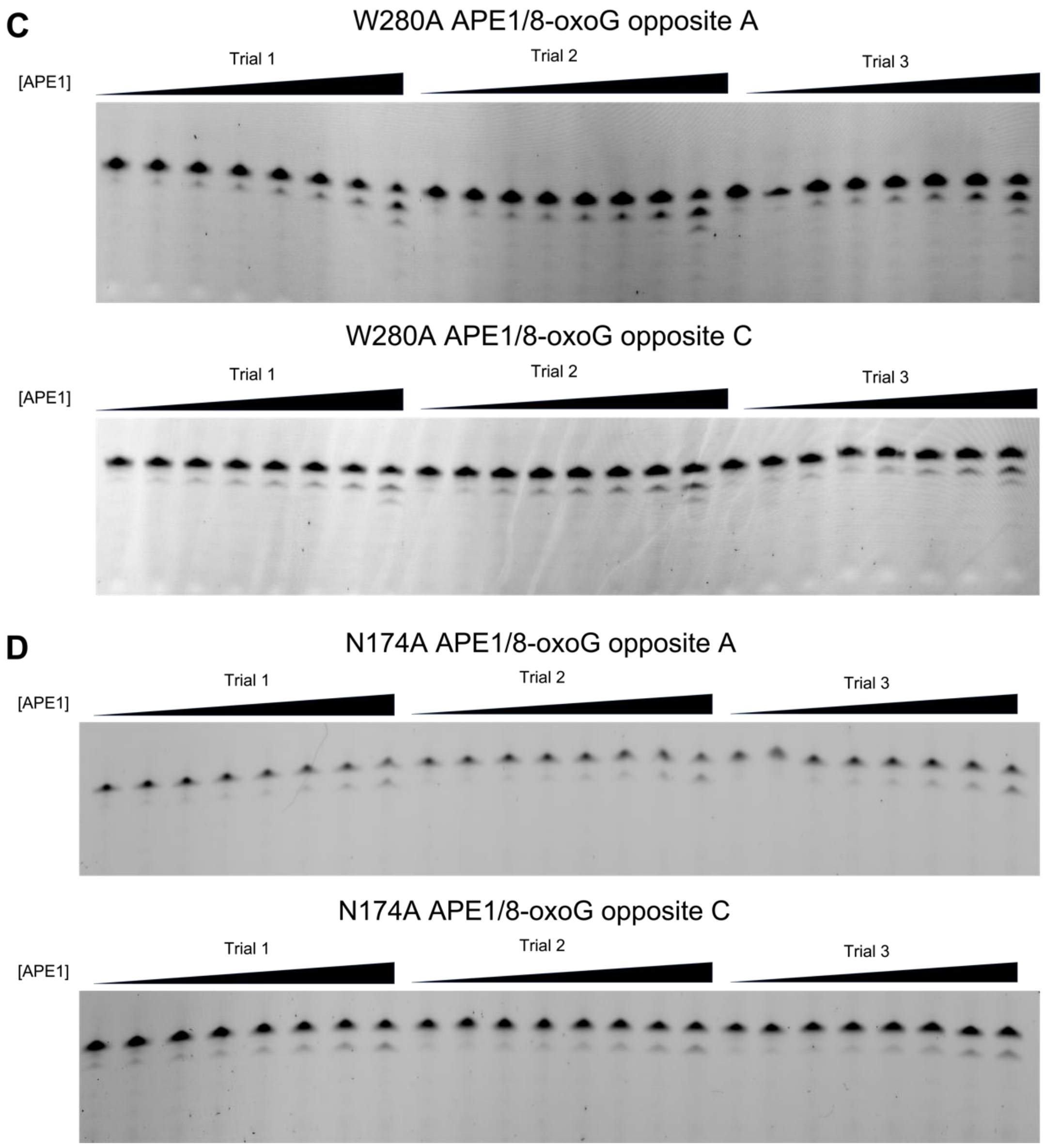
C
W280A APE1/8-oxoG opposite A
[APE1]
Trial 1
Trial 2
Trial 3
W280A APE1/8-oxoG opposite C
[APE1]
Trial 1
Trial 2
Trial 3
D
N174A APE1/8-oxoG opposite A
[APE1]
Trial 1
Trial 2
Trial 3
N174A APE1/8-oxoG opposite C
[APE1]
Trial 1
Trial 2
Trial 3

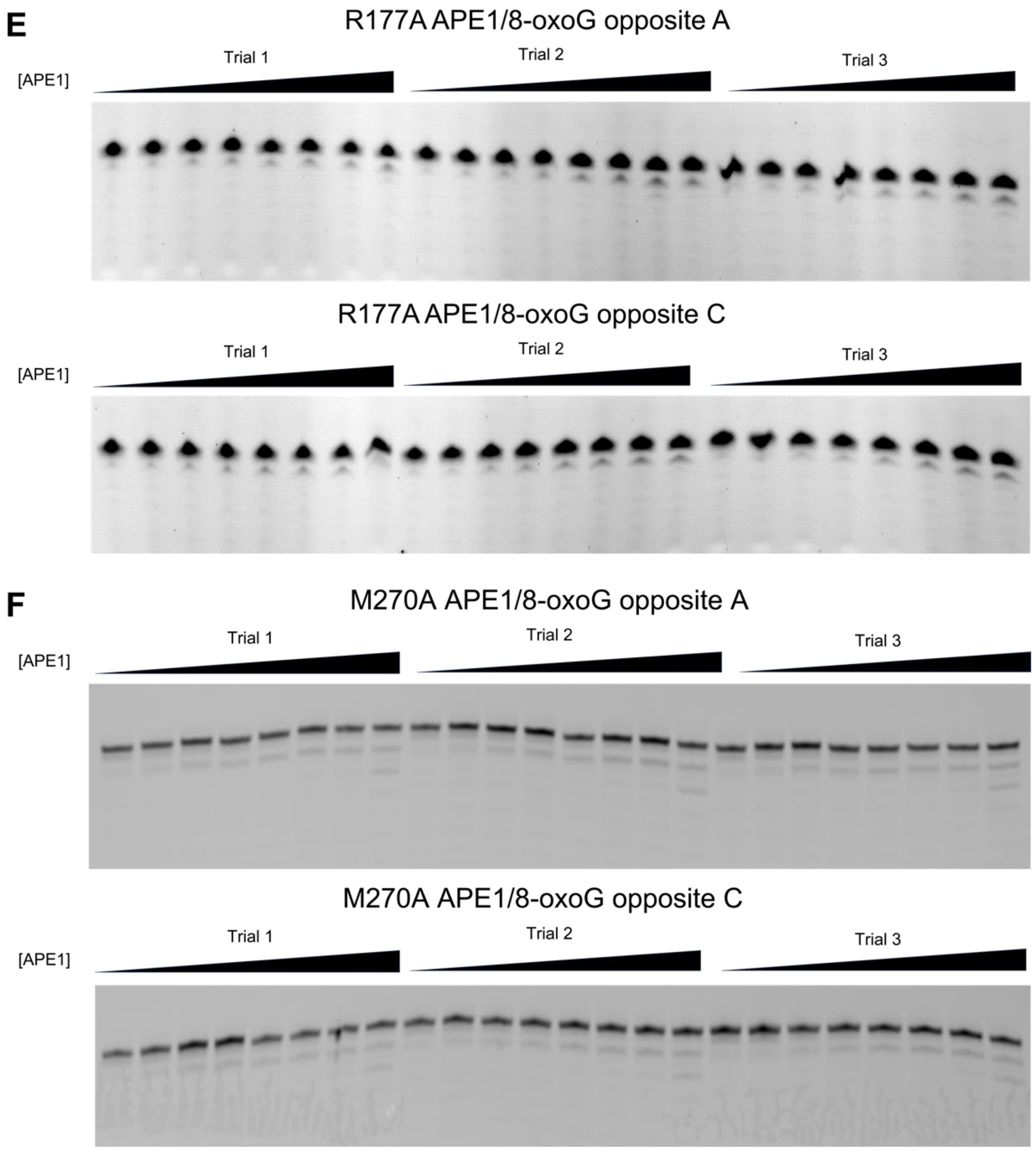

Supplemental Figure 3: Gel images used to calculate APE1 product formation. A) WT APE1 B) F266A APE1 C) W280A APE1 D) N174A APE1 E) R177A APE1 and F) M270A APE1. DNA substrates are indicated, and each trial included a range of APE1 concentrations of 0, 0.5, 2.5, 5, 12.5, 25, 50, and 250 nM.
